# The cadmium-responsive gene, *cdr-6*, does not influence *Caenorhabditis elegans* responses to exogenous zinc

**DOI:** 10.17912/micropub.biology.000305

**Published:** 2020-09-14

**Authors:** Kathryn S Evans, Erik C. Andersen

**Affiliations:** 1 Molecular Biosciences, Northwestern University, Evanston, IL 60208; 2 Interdisciplinary Biological Sciences Program, Northwestern University, Evanston, IL 60208; 3 Robert H. Lurie Comprehensive Cancer Center, Northwestern University, Chicago, IL 60611

**Figure 1:**
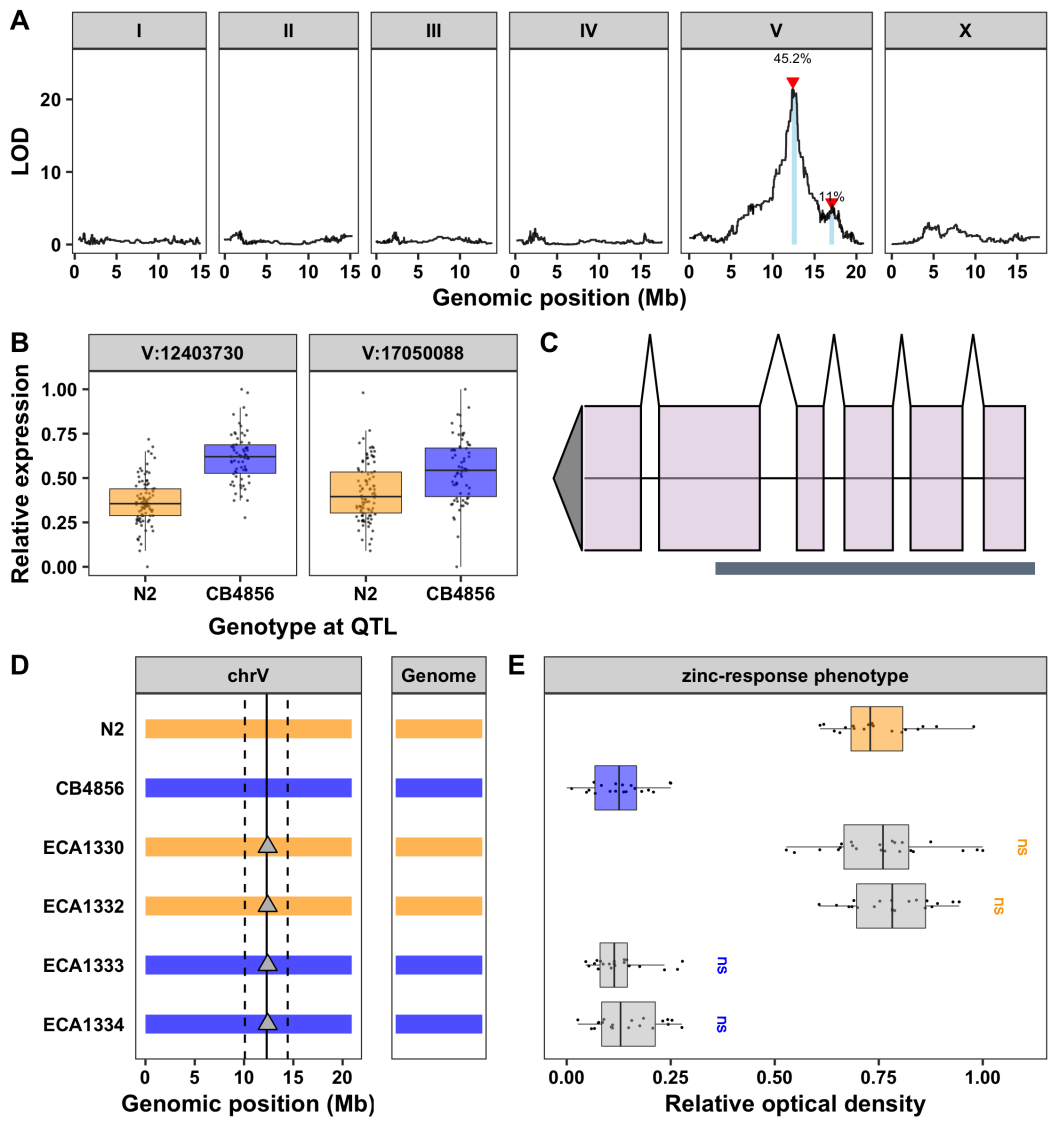
**Testing the role of *cdr-6* in the *C. elegans* zinc response.** **A)** Results from linkage mapping analysis using expression of *cdr-6* as a quantitative trait. Genomic position (x-axis) is plotted against the logarithm of the odds (LOD) score (y-axis) for 13,003 genomic markers. Each significant QTL is indicated by a red triangle at the peak marker, and a blue rectangle shows the 95% confidence interval around the peak marker. The percentage of the total variance in the RIAIL population that can be explained by each QTL is shown above the QTL. **B)** For each QTL, the expression of *cdr-6* (y-axis) in RIAILs split by genotype at the marker with the maximum LOD score (x-axis) are plotted as Tukey box plots. Each point corresponds to the relative expression of a unique recombinant strain. Strains with the N2 allele are colored orange, and strains with the CB4856 allele are colored blue. **C)** Gene model for *cdr-6* is shown. Exons are represented by light purple rectangles and introns are represented by connecting lines. Location of the CRISPR-Cas9 deletion is shown with the grey box below the gene model. **D)** Strain genotypes are shown as colored rectangles (N2: orange, CB4856: blue) in detail for chromosome V (left) and in general for the rest of the chromosomes (right). The solid vertical line represents the peak marker of the zinc-response QTL, and the dashed vertical lines represent the confidence interval. Grey triangles represent *cdr-6* deletions. **E)** Relative animal optical density in zinc (median.EXT, x-axis) is plotted as Tukey box plots against strain (see C, y-axis). Statistical significance of each strain compared to its parental strain (ECA1330 and ECA1332 to N2 and ECA1333 and ECA1334 to CB4856) is shown above each strain and colored by the parent strain it was tested against (ns = non-significant, *p-*value > 0.05; * = significant, *p*-value < 0.05).

## Description

Regulation of the essential trace element zinc is necessary to avoid the toxic consequences caused by too little or too much of this metal (Vallee and Falchuk 1993; Rosen 2006). The zinc-response pathway has been extensively studied in the nematode roundworm *Caenorhabditis elegans* and several genes have been discovered that function to modulate sensitivity to both high and low zinc concentrations (Dietrich **et al.** 2016). Recently, we identified a quantitative trait locus (QTL) on the center of chromosome V, indicating that natural genetic variation between the laboratory strain, N2, and a genetically divergent wild isolate from Hawaii, CB4856, contributes to differential responses to excess zinc (Evans **et al.** 2020).

The confidence interval for this QTL is 1.6 Mb and contains 629 genes (WS263). Of these genes, 113 have one or more genetic variants predicted to modify the amino-acid sequence of the protein. However, protein-coding variation is just one of the ways that genetic variation can cause phenotypic variation. Another is variation in gene expression, which is hypothesized to be important in the majority of complex traits (Hindorff **et al.** 2009). We identified 83 expression QTL (eQTL) within this 1.6 Mb region using the eQTL dataset that mapped expression differences among a panel of recombinant inbred advanced intercross lines (RIAILs) also derived from N2 and CB4856 (Rockman **et al.** 2010; Evans and Andersen 2020). The most significant eQTL in this region caused a change in expression of the gene *cdr-6* (**Figure 1A**). This gene, a homolog of the cadmium-response gene *cdr-1*, is highly expressed in intestinal and pharynx muscle cells during larval stages and is downregulated after treatments with arsenic, cadmium, or zinc (Dong **et al.** 2008). Furthermore, *cdr-1* was previously shown to mitigate cadmium toxicity in *C. elegans* (Dong *et al.* 2005; Hall *et al.* 2012). Together, these data suggest that expression of *cdr-6* might be toxic to *C. elegans* in the presence of heavy metals. Additionally, RIAILs with the CB4856 allele on chromosome V naturally express higher levels of *cdr-6* (**Figure 1B**) and are also more sensitive to zinc than RIAILs with the N2 allele (Evans **et al.** 2020). This result indicates that strains with naturally low expression of *cdr-6* are more resistant to excess zinc than strains with naturally high expression of this gene.

To test this hypothesis, we used CRISPR-Cas9 genome editing to create strains with large deletions of *cdr-6* in both the N2 and CB4856 genetic backgrounds (**Figure 1C,D**). Because expression of *cdr-6* was higher in RIAILs with the CB4856 allele (associated with zinc sensitivity) than in RIAILs with the N2 allele (associated with zinc resistance) (**Figure 1B**), we expected that a knockout of *cdr-6* in the CB4856 genetic background would cause increased resistance to excess zinc. Alternatively, if variation in expression of *cdr-6* underlies the zinc-response QTL on chromosome V, a knockout of *cdr-6* in the N2 genetic background should not cause an increase in zinc resistance. We exposed N2, CB4856, and two strains with independently derived *cdr-6* deletion alleles in each genetic background to elevated zinc and measured their optical densities using a high-throughput assay with the COPAS BIOSORT (Andersen **et al.** 2015; Evans and Andersen 2020; Evans **et al.** 2020). We found that strains with a deletion of *cdr-6* phenocopied the strain with the same genetic background (**Figure 1E**), suggesting that differences in expression of *cdr-6* do not underlie zinc responses.

This study not only provides evidence against *cdr-6* as the causal gene underlying differences in zinc resistance between the N2 and CB4856 strains, but also indicates that *cdr-6* does not influence zinc resistance in *C. elegans*. Although a previous study showed that cdr-6 was downregulated in response to zinc (Dong **et al.** 2008), our results do not contradict theirs. In fact, the authors also showed that the accumulation of fluid-filled droplets in the pseudocoelom, a phenotype observed in strains with inhibited CDR-6 expression using RNAi, is not increased in response to zinc or cadmium exposure (Dong **et al.** 2008). Taken together, we conclude that expression of *cdr-6* decreases in response to zinc but animal development in the presence of zinc is not affected by *cdr-6* function. It is likely that *cdr-6* does not function in the nematode zinc response but rather is downregulated as an indirect effect of zinc exposure.

## Methods

**Strains**

Animals were grown at 20°C on modified nematode growth media (NGMA) containing 1% agar and 0.7% agarose to prevent burrowing and fed OP50 (Andersen **et al.** 2014). The *cdr-6* deletion strains are described below. All strains are available upon request.

**Table:** 

**Strain**	**Genotype**	**Deletion**
ECA1330	*cdr-6(ean181)* [N2 background]	730 bp deletion from V:12,413,681-12,412,951
ECA1332	*cdr-6(ean183)* [N2 background]	811 bp deletion from V:12,413,723-12,412,912
ECA1333	*cdr-6(ean184)* [CB4856 background]	632 bp deletion from V:12,413,034-12,413,665
ECA1334	*cdr-6(ean185)* [CB4856 background]	788 bp deletion from V:12,412,864-12,413,651

**Expression QTL mapping**

Microarray expression for 14,107 probes were previously collected from 208 RIAILs (Rockman and Kruglyak 2009; Rockman **et al.** 2010), filtered (Andersen **et al.** 2014), and mapped using linkage mapping with 13,003 SNPs (Brady **et al.** 2019; Evans and Andersen 2020). The probe that measures expression for *cdr-6* is A_12_P101644. All expression and eQTL data can be found in Evans and Anderson, 2020.

**Generation of *cdr-6* deletion strains**

Deletion alleles for *cdr-6* were generated using CRISPR-Cas9 genome editing as previously described (Hahnel **et al.** 2018; Brady **et al.** 2019). Briefly, 5’ and 3’ guide RNAs (Synthego, Redwood City, CA) were designed with the highest possible on-target and off-target scores (Doench **et al.** 2016). The CRISPR injection mix (1 µM *dpy-10* sgRNA, 5 µM of each sgRNA for *cdr-6*, 0.5 µM of a single-stranded oligodeoxynucleotide template for homology-directed repair of *dpy-10* (IDT, Skokie, IL), and 5 µM Cas9 protein (Q3B Berkeley, Berkeley, CA) in water) was assembled and incubated for an hour before injection. Young adults were mounted onto agar injection pads, injected in either the anterior or posterior arm of the gonad, and allowed to recover on 6 cm plates. Survivors were transferred to individual 6 cm plates 12 hours later and allowed to lay embryos. After two to three days, the F1 progeny were screened and individuals with Rol or Dpy phenotypes were selected and transferred to new 6 cm plates. After 48 hours, the F1 individuals were genotyped by PCR flanking the desired deletions. Individuals with deletions were propagated and genotyped for at least two additional generations to ensure homozygosity and to cross out the Rol mutation. Deletion amplicons were Sanger sequenced to identify the exact location of the deletion. Reagents used to generate and validate the deletion are found below:

crECA36 *dpy-10* guide RNA:

GCUACCAUAGGCACCACGAG

crECA37 *dpy-10* repair construct: CACTTGAACTTCAATACGGCAAGATGAGAATGACTGGAAACCGTACCGCATGCGGTGCCTA GGTAGCGGAGCTTCACATGGCTTCAGACCAACAGCCTAT

*cdr-6* guide RNA:

crECA153: TCCAGTGACAACAACCCTAG

crECA115: AGTTTATAGTCGTTGTGTTG

External primers for deletion validation:

oECA1635: ACACACGTTCACTGGCTAGACT

oECA1636: AGCACGTCGTTGATATGCGAAC

Internal primers for deletion validation:

oECA1365: ACACACGTTCACTGGCTAGACT

oECA1637: TGTGTTCCCCATTGAGCTCGAT

**High-throughput zinc-response assay**

Zinc response was measured as a function of animal optical density (representing nematode development) as described previously (Brady **et al.** 2019; Evans and Andersen 2020). Briefly, strains were propagated for two generations, bleach-synchronized, and titered in K medium (Boyd **et al.** 2012) at a concentration of 25-50 embryos per well of a 96-well microtiter plate. The following day, arrested L1s were fed HB101 bacterial lysate (Pennsylvania State University Shared Fermentation Facility, State College, PA; (García-González **et al.** 2017)) at a final concentration of 5 mg/mL with either 1% water or 250 µM zinc in 1% water. Nematodes were allowed to grow at 20°C with constant shaking for 48 hours, treated with sodium azide (5 mM in water), and analyzed using the COPAS BIOSORT (Union Biometrica; Holliston, MA). Animal optical densities collected by the BIOSORT were processed and analyzed using the R package *easysorter* (Shimko and Andersen 2014) as described previously (Brady **et al.** 2019). Each strain has a minimum of 20 replicates and each replicate is defined by the median animal optical densities of six to 32 animals. Pairwise tests for statistically significant differences in the zinc response between strains were performed using the *TukeyHSD* function (R Core Team 2017) on an ANOVA model with the formula *(phenotype ~ strain)*. For plotting purposes, these residual values were normalized from zero to one with zero being the well with the smallest value and one the well with the largest value.
